# Outcome following emergency surgery for refractory severe ulcerative colitis in a tertiary care centre in India

**DOI:** 10.1186/1471-230X-5-39

**Published:** 2005-11-30

**Authors:** Sujoy Pal, Peush Sahni, Girish K Pande, Subrat K Acharya, Tushar K Chattopadhyay

**Affiliations:** 1 Department of GI Surgery & Liver Transplantation, All India Institute of Medical Sciences, Ansari Nagar, New Delhi-110029, India; 2 Department of Gastroenterology & Human Nutrition Unit, All India Institute of Medical Sciences, Ansari Nagar, New Delhi-110029, India

## Abstract

**Background:**

Steroid-based intensive medical therapy for severe ulcerative colitis is successful in 60–70% of such patients. Patients with complications or those refractory to medical therapy require emergency colectomy for salvage. Little is known about the impact of timing of surgical intervention and surgical outcomes of such patients undergoing emergency surgery in India where the diagnosis is often delayed or missed in patients who are poor, malnourished and non-compliant to medical treatment.

**Methods:**

The clinical records of all patients undergoing emergency surgery for severe ulcerative colitis or its complication in the Department of GI surgery AIIMS, New Delhi, India, between January 1985 and December 2003 were retrieved and data pertaining to demographic features, duration of intensive medical therapy, presence of complications, time from admission to emergency surgery, surgical procedure, in-hospital morbidity and mortality and follow up status extracted.

**Results:**

A total of 72 patients underwent emergency surgery (Subtotal colectomy: 60; ileostomy alone under local anaesthesia: 12). Poor nutritional status was seen in 61% of the patients.

Twenty-one patients (29%) underwent emergency surgery for complications of severe ulcerative colitis such as colonic perforation (spontaneous 6, iatrogenic 4), massive lower gastrointestinal haemorrhage (5), toxic megacolon (4) and large bowel obstruction (2). The remaining patients (n = 51) underwent emergency surgery following failed intensive therapy; 17 underwent surgery ≤5 days (Group I) and 34 were operated >5 days (Group II) after initiation of intensive therapy. In this group all the post-operative deaths (n = 8) occurred in those who were operated after 5 days. The difference in mortality in these two groups (i.e. surgical intervention ≤ or >5 days) was statistically significant {0/17 (Group I) vs 8/34 (Group II); p = 0.03}.

Overall, 12 patients died (in-hospital mortality: 16.7%). The mortality was higher (10/43; 23.3%) in our early experience (i.e. 1985–1995) when compared to our subsequent experience (2/29; 6.9%) (1996–2003).

A total of 48 patients (including 3 awaiting a restorative procedure) are alive on follow up (66.7%; 3 patients lost to follow up). A restorative procedure could be successfully completed in 81% of the survivors of the emergency procedure.

**Conclusion:**

To optimize the outcome, a combined team of physicians and surgeons should be involved in the management of patients with severe ulcerative colitis with focus on nutritional support, correction of metabolic derangements, close clinical monitoring and timely assessment for the need for emergency surgery. This retrospective analysis shows that improved results can be achieved with experience and by following a policy of early surgical intervention within 5 days, especially in patients who have failed intensive medical therapy.

## Background

It is known that about 20% of patients with ulcerative colitis, suffer from severe acute relapse and its associated complications at some point in the course of their illness [1,2]. Overall, 5–10% of patients with ulcerative colitis present with a severe first attack. The relapse-free interval in a given patient remains unpredictable. However, long-term prospective cohort studies have shown that virtually all patients will relapse and develop acute exacerbation at some point in the course of their illness [1,2]. Intensive medical treatment (Oxford regimen) is successful in 60%–70% of patients suffering from severe ulcerative colitis, but the rest require emergency surgery either for a complication or because they fail to respond to medical therapy [3-6]. Among those who respond to intensive medical therapy, a colectomy will be required in up to 50% within a year and in at least 75% at around 5 years of follow up [6,7]. Emergency surgery in anaemic, nutritionally depleted, immunosuppressed and toxic patients has a high morbidity and mortality [8,9]. Little is known about the outcome of patients with acute severe ulcerative colitis undergoing emergency surgery in India. We hypothesized that Indian patients with ulcerative colitis may behave differently because they are poor (have difficulty in purchasing drugs) and often illiterate (do not understand the importance of continuing medication and follow up) and the diagnosis is often delayed because of the high incidence of infective diarrhoea and dysentery. We therefore analyzed our experience of emergency surgery in patients with ulcerative colitis focusing on two main issues, i.e. the timing of surgery and the immediate as well as long term outcome following emergency surgery.

## Methods

All patients undergoing emergency surgery for severe ulcerative colitis or its complication in the Department of GI Surgery, All India Institute of Medical Sciences, New Delhi, between January 1985 and December 2003 had a pre-designed proforma filled. The details recorded included demographic features of the patients, duration of intensive medical therapy during the index admission, presence of complications (toxic megacolon, perforation, lower gastrointestinal haemorrhage, intestinal obstruction and colorectal cancer), time from initiation of intensive therapy to emergency operation, indication for emergency surgery, type of surgical procedure, postoperative complications, in-hospital mortality, delayed deaths during follow up, outcome of any further operative procedures and current status. The collected data was retrospectively analyzed.

A severe episode of ulcerative colitis was defined according to Truelove and Witt's criteria [3] as: >6 bloody stools/day, fever ≥ 38°C, tachycardia > 100/min, anemia and/or an erythrocyte sedimentation rate > 30 mm in 1^st ^hour.[3]

All patients diagnosed to have severe ulcerative colitis were admitted in the Gastroenterology department of our institution and were managed jointly, from the time of the admission, by a team comprising of physicians and surgeons. Nutritional status at admission was judged by a combination of subjective clinical assessment, body weight record (labeled underweight when <10^th ^percentile of their expected weight as per sex and height) and serum albumin levels <3.0 g/dl was considered low. Intensive medical therapy included parenteral steroids (100 mg hydrocortisone intravenously 6 hourly), nil by mouth, nasogastric aspiration, intravenous crystalloids and electrolytes, parenteral nutritional support, blood transfusions and broad spectrum parenteral antibiotics (third-generation cephalosporins, aminoglycosides and metronidazole). The indications for emergency surgery included lack of response to or deterioration while on intensive medical therapy and presence of complications at admission. Patients who continued to have high stool frequency (>6 motions/day), persistent hematochezia, deteriorated clinically, developed steroid toxicity and/or complications and/or remained intolerant to oral feeding were judged to have failed to respond to intensive medical therapy. In our practice (before this study), duration of intensive therapy did not exclusively influence the decision to operate or label a patient refractory to intensive therapy.

### Operative procedures

A subtotal colectomy with Hartmann's pouch (STC) was done in most patients. This included removal of the diseased ascending, transverse, descending and the sigmoid colon with closure of the sigmiodorectal stump at or above the level of sacral promontory without any pelvic mobilization of the rectum. When the rectal stump was unhealthy because of severe rectal disease or when there was ongoing rectal bleeding the open end was brought out as a mucus fistula through the anterior abdominal wall. Some patients underwent an initial loop ileostomy under local anaesthesia, as they were considered too ill to withstand a colectomy under general anaesthesia. Subsequently, in the same hospital admission or at an early readmission these patients underwent a subtotal colectomy.

All patients were counseled regarding the long-term benefits/disadvantages of a restorative proctectomy with an ileal pouch (J reservoir) anal anastomosis (IPAA) [10,11]. Depending on their motivation and acceptance a restorative procedure with a diverting loop ileostomy was then done 3–6 months after the emergency colectomy. Finally, 6 weeks later, the ileostomy was closed after obtaining a pouchogram.

### Follow up protocol

Patients were followed up at 3-monthly intervals in the first year, 6-monthly intervals in the second year and at yearly intervals thereafter. Patients who defaulted were sent postal reminders and questionnaires to ascertain their current status. At each visit the patients underwent a general physical examination, their weight was noted, laparotomy wound and ileostomy site were inspected and patients who had had the ileostomy closed were interviewed regarding their bowel frequency and continence. Periodic complete blood counts and liver function tests were also done.

The primary outcome measure was operative mortality. The factors which could have influenced the patient outcome such as duration of disease, long term use of steroids or azathioprine, presence of fever, co morbid illnesses, preoperative hemoglobin, total leucocyte count and serum albumin levels were also recorded. We also analyzed the surgical outcome in relation to duration of intensive medical management in the index admission. For this purpose we divided the patients who were considered failures of intensive medical therapy into 2 groups: those who underwent surgery ≤ and >5 days after the initiation of intensive medical therapy.

### Statistical methods

The information was entered in a MS Access format and analyzed using the SPSS version 11.5 software. For comparison, non-parametric tests such as Pearson's chi square test and for continuous variables student's t test were used. A *p *value less than 0.05 was considered significant. A univariate analysis was done to determine any association of operative mortality with duration of intensive medical therapy, age, preoperative nutritional status duration of disease, long term use of steroids, presence of fever, co morbid illnesses, preoperative hemoglobin, total leucocyte count and serum albumin levels. To identify a cut-off point with regard to duration of intensive medical therapy a receiver-operator characteristic (ROC) curve was plotted and the point on the curve giving the highest sensitivity for the primary outcome measure(postoperative death) was used for the subsequent analysis.

## Results

Between 1985–2003, 154 (88 males) patients underwent operations for ulcerative colitis out of whom 72 (46.5%) underwent emergency surgery. The mean age was 34.8 ± 12.8 years (range: 14–72 years; median: 32 years). The nutritional status of 61% of these patients was poor and the mean serum albumin level was 2.6 g/dl (range: 1.3–4.8 g/dl). Failure of intensive medical therapy was the most common indication for emergency surgery (n = 51; 71%) patients. In these patients the interval between initiation of intensive medical therapy at our hospital and emergency surgery varied between 3–35 days with a mean of 9 days (median: 6 days). The remaining 21 (29%) patients had presented with complications such as colonic perforation (spontaneous 6, iatrogenic 4), massive lower gastrointestinal haemorrhage (5), toxic megacolon (4) and large bowel obstruction (2). A majority of these patients underwent urgent/emergent surgery even before a trial of intensive drug therapy could be initiated.

### Surgery

The most common emergency surgical procedure was a sub total colectomy with a Hartmann's pouch or mucus fistula (n = 60; 83.3%). A diverting loop/divided ileostomy alone (under local anaesthesia) was the initial procedure in 12 patients. Ileostomy was more frequently used as the initial procedure in the later period of our experience (i.e., 1996–2003; 10/29 patients vs. 2/43; p = 0.003).

### Postoperative morbidity

The overall morbidity included major wound sepsis with or without wound dehiscence (27; 37.5%), fever (20; 27.8%), adhesive intestinal obstruction (8.3%), pelvic sepsis (6.6%), ileostomy-related complications (6.6%), blow out of Hartmann's pouch (6.6%) and continued rectal bleeding (3.3%). The median duration of postoperative hospital stay was 10 days.

### Postoperative mortality

Overall, 12 (STC: 10/60; Ileostomy: 2/12) patients died postoperatively in the same admission giving an in-hospital mortality of 16.7%. Eight patients died in the sub group operated following failure of intensive medical therapy (8/51; 15.7%) whereas 4 died following surgery for complications (4/21; 19%). Notably, all patients with perforations alone (n = 10) survived the emergency surgery.

The causes of death included septicaemia in 6, severe chest infection with respiratory failure in 2, disseminated intravascular coagulation in 1, diabetic ketoacidosis in 1, metabolic encephalopathy in 1 and upper gastrointestinal bleeding (stress bleeding) in 1. By using chi square tests it was found that the risk of death increased in those patients who had postoperative fever (p = 0.01), ileostomy-related complications (p = 0.02) or evidence of peritonitis at surgery (p = 0.018). Also, the patients who died had lower preoperative albumin levels (mean albumin: 2.25 ± 0.5 g/dl vs 2.73 ± 0.8 g/dl; p = 0.08).

We also found that the mortality was higher (10/43; 23.3%) in our early experience (i.e. 1985–1995) when compared to our subsequent experience (2/29; 6.9%) (1996–2003).

### Timing of surgery

Twenty-one patients had emergency surgery for complications and in 51 patients the indication was failure of intensive medical therapy. In the 21 patients undergoing surgery for complications the occurrence of the complication determined the timing of the surgery. In this group, often surgery was done shortly after admission (i.e. in patients with perforation, severe bleeding or obstruction).

On analyzing the data of the patients who underwent emergency surgery for failed intensive therapy (n = 51), statistical analysis with regard to mortality as the primary outcome measure showed that the only variable that influenced the outcome significantly was the timing of surgery from the initiation of intensive medical therapy. A cut-off time period of 5 days was obtained after plotting a ROC curve with death as the primary outcome measure. In this group 17 patients underwent surgery ≤ 5 days and the rest (n = 34) were operated >5 days after initiation of intensive therapy. The operative outcome of each group was further analyzed. All the post-operative deaths (n = 8) occurred in those who were operated after 5 days (Table 1). The difference in mortality in the two groups based on the timing of surgery (i.e. ≤ or >5 days) was statistically significant {0/17 (Group I) vs 8/34 (Group II); p = 0.03}. These two groups were otherwise comparable in terms of the nutritional status, age, sex, and associated co-morbidities. Table 2 summarizes the salient clinical, demographic and laboratory data pertaining to these two groups of patients who failed intensive medical therapy. The groups did not show any significant difference in any of the variables compared.

### Long term outcome

Two patients died at home (6 weeks; 8 weeks) after being discharged alive from hospital of which one patient who had severe associated comorbidity in the form of valvular heart disease with infective endocarditis, died of a suspected cerebral thromboembolic phenomenon and another patient died of disseminated colorectal cancer.

**Figure 1:**
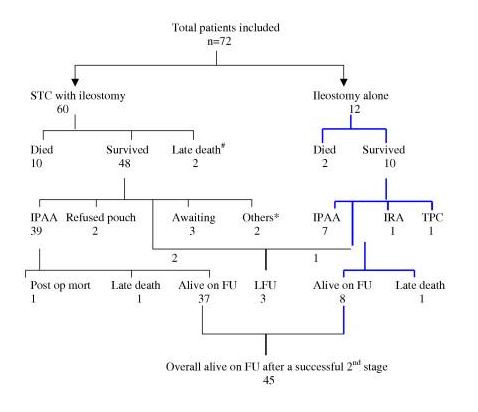
**Flow chart showing the procedures done and outcome (short and long term)**. IPAA: ileal J Pouch anal anastomosis; IRA: ileorectal anastomosis; TPC: total proctocolectomy with permanent Brooke's ileostomy; FU: follow up; LFU: lost to follow up. * One patient had rectal cancer receiving adjuvant therapy and IPAA was not contemplated; the other patient developed intestinal tuberculosis and recurrent small bowel obstruction with multiple reoperations, hence pouch was not advocated. # One patient died of massive cerebral thromboembolism secondary to valvular heart disease with infective endocarditis; one additional patient died of disseminated colorectal cancer.

The ultimate outcome of each patient presenting to us for emergency surgery is summarized in Figure 1 and Table 3. All surviving patients have been followed up for a mean period of 39 months (range: 3–216 months). Of the 58 (12 postoperative and 2 late deaths) patients who survived the emergency procedure, 3 patients were lost to follow up, 2 refused a second stage IPAA and 2 were not offered because of extensive small bowel tuberculosis in one and low rectal cancer in another. Hence, 51 patients were available for a 2^nd ^stage restorative proctectomy. In these, IPAA was done in 46 patients, one patient is alive and well following an ileorectal anastomosis (IRA), one other patient is alive and well following a total proctocolectomy (TPC) with Brooke's ileostomy (a 65 year old patient who had poor anal sphincter tone opted for a TPC) and 3 are awaiting a restorative surgery. One patient died following the pouch procedure and there were 2 late deaths on long term follow up.

A total of 48 patients (including 3 awaiting a restorative procedure) are alive on follow up, from the initial cohort of 72 patients (66.7%; status of 3 patients lost to follow up not known). The restorative procedure (IPAA, IRA, TPC) could be successfully completed in 81% of the survivors of the emergency procedure (47/58 with 1 postoperative death). Of these 45 patients are alive and doing well.

## Discussion

Before 1950, the expected mortality of a patient with acute severe ulcerative colitis was as high as 40–50% [1-3]. Such a patient was a physician's nightmare, as the surgeons never came in the picture. Truelove and Witts introduced steroid therapy in the 1950s [3,4]. With this, the mortality decreased to 8–10%, but rates as high as 18% have been reported [4-6]. The last milestone in therapy came in the 1960's after Brooke and Sampson proposed emergency total colectomy for salvage of patients following failure of medical therapy [12].

The timing of the emergency surgery is of crucial importance for these patients, as procrastination leads to worsening of the patient's general condition and nutritional status. To begin with, nearly two-thirds of the patients in this series were poorly nourished and hypoalbuminemic before surgery. It has been shown previously that the surgical outcome worsens with delay beyond 5–7 days of intensive medical therapy [8,13]. Our data supports this view, as the operative mortality in the group of patients who failed intensive medical therapy and were operated late (i.e., beyond 5 days) was high when compared to the group operated within 5 days (8/34 *vs*. 0/17; p = 0.03). In fact, all the post-operative deaths (n = 8) occurred in patients who were operated on after 5 days. This difference in mortality in the two groups based on timing of surgery is statistically significant. A univariate analysis of this group could not identify any other significant risk factor for mortality (Table 2). In 46 patients (i.e., 64% of the total patient population n = 72), multiple factors were responsible for a delay >5 days in surgical intervention. Some patients had shown an initial response to intensive medical therapy but had a relapse or developed a complication in the same hospitalization; some had associated comorbid conditions in the form of heart disease, uncontrolled diabetes or tuberculosis while some were referred late from other hospitals.

In patients who present with complications such as perforation or massive colonic bleeding, emergency surgery should be performed as soon as possible. In patients with toxic megacolon a short period of conservative management (24–48 hours) may be beneficial [2,14]. In patients with large bowel obstruction usually 48–72 hours of preoperative preparation is necessary to optimize the surgical outcome. Similar to other reports in the literature, patients with a combination of toxic megacolon, perforation and fecal peritonitis had the poorest outcomes [2,8,14].

On analyzing the cause of death (pertaining to the entire study group) we found that 8 patients died of septicaemia, the remaining 4 had severe metabolic disturbances with encephalopathy, coagulopathy and respiratory failure from which they could not be salvaged. A combination of severe metabolic derangement along with pre existing sepsis (fecal peritonitis) or coagulopathy was lethal. In only one of the patients who died due to the metabolic sequelae (diabetic ketoacidosis) of pre-existing insulin-dependent diabetes mellitus, death could be attributed to comorbidity. As mentioned earlier, the overall postoperative mortality was higher (10/43; 23.3%) in our early experience (i.e. 1985–1995) when compared to our subsequent experience (2/29; 6.9%). We can only hypothesize that this improvement in operative outcome was in part due to the institution of an aggressive medical management protocol (following 1996) with *early *parenteral nutrition and emphasis on correction of metabolic derangements preoperatively. Another factor that could have improved the results was the conscious decision to use a decompressing ileostomy alone, as the first stage procedure, in very sick and moribund patients (Post-1996:10/29; 34.5% *vs *Pre-1996: 2/43; 4.7%; p = 0.003). Although the data is limited, we believe that this approach allows us to buy time and improve the patients overall condition so that the patient is able to withstand colectomy at a later date. Our current mortality figures are comparable to most other western series [8,9,15].

Following sub total colectomy (STC), the commonest morbidity was related to wound sepsis and postoperative fever. Patients with peritonitis at operation, those who developed postoperative fever or had ileostomy related complications were more likely to die postoperatively. In our experience there were 4 (6.6%) instances with stump 'blow outs' which caused pelvic sepsis and abdominal wound dehiscence and led to a prolonged hospital stay. Bleeding from the Hartmann's pouch did occur in a few patients (3.3%), but was managed with a combination of mesalamine and steroid enemas, local adrenaline saline lavages or by tapering the steroids slowly. A Hartmann's procedure or a mucus fistula of the rectal stump has been recommended by other workers also [16].

In the emergency setting, it is believed that a subtotal colectomy is the best option as it is technically simple, avoids dissection in the pelvis, minimizes blood loss and allows a subsequent restorative pouch procedure [8,9,15,17]. It also allows a definitive histological diagnosis to be obtained. In our study, all patients who had STC and survived (n = 60), were weaned off steroids after a month. Fifty-eight patients (2 late deaths) recovered fully and gained weight. Out of the 56 patients finally considered eligible for a second stage IPAA procedure, 47 underwent a second stage procedure (IPAA: 46; IRA: 1) with 1 postoperative death following an IPAA (Figure 1 and Table 3). The details of the patients' management have been listed in the flow chart (Figure 1). The second stage could be successfully performed in 81% (47/58) of the cases who survived the first procedure. Notably, by the time the patients undergo the 2^nd ^stage procedure, the postoperative results obtained are similar to what can be achieved by an elective restorative proctocolectomy done in a patient with long-standing disease. Our second stage operative mortality was low at 2.1% (1/47) and comparable to the mortality rate in the elective setting (1/69; 1.5%; unpublished data). From the initial cohort of 72 patients, 48 patients are alive on follow up. Hence, two-third of the patients have been successfully salvaged and rehabilitated by staged surgery for severe ulcerative colitis. It is worth emphasizing here that the long term results of salvage and rehabilitation reflect the survival rate following the initial emergency surgery, as is apparent from our experience. This attrition following the first stage surgery is often unavoidable in 10–15% of cases because of late deaths, incidental detection of malignancy/Crohn's or indeterminate colitis [17], refusal for further surgery, associated intestinal/miliary tuberculosis and other co-morbid illnesses.

Based on this experience we feel that in order to achieve a good postoperative outcome a combined team of physicians and surgeons should be involved in the management of patients with severe ulcerative colitis right from the day of admission. Aggressive nutritional therapy and correction of metabolic derangements combined with close clinical monitoring should be instituted along with the intensive steroid regimen [18-20]. Need for surgery should be assessed in advance [7,21] and early surgery is recommended in the event of failed medical treatment. In a majority of such cases a sub total colectomy is the procedure of choice and gives the best results. A loop ileostomy alone can be used in severely ill and moribund patients to buy time for a subsequent colectomy. These measures, when strictly implemented, would help to achieve a low and acceptable mortality rate.

In India, emergency surgery for salvaging ulcerative colitis will continue to play an important role as few medical centers have the experience and resources to treat such patients with optimal skill and judgment, and nearly a third of the patients are refractory to intensive medical therapy. In consonance with the experience of other western centers [8,9,13], our retrospective analysis has reinforced that improved results can be achieved with experience and by following a policy of early surgical intervention within 5 days, especially in patients who have failed intensive medical therapy. Since immediate operative outcome is linked to the timing of the surgery, emphasis on anticipating failures of intensive medical therapy early is crucial for optimal decision-making and successful rehabilitation of these patients.

## Conflicts of interest

The author(s) declare that they have no competing interests.

## Authors' contributions

*Sujoy Pal*: Planning, data collection, study design and analysis, surgical management of patients, drafting and revising the manuscript

*Peush Sahni*, *Girish K. Pande*: Planning, study design, surgical management of patients included, drafting and revising the manuscript

*SK Acharya*: Planning, study design, medical management of patients, drafting and revising the manuscript

*TK Chattopadhyay*: Planning, study design, surgical management of patients, drafting and revising the manuscript

## Pre-publication history

The pre-publication history for this paper can be accessed here:



## Figures and Tables

**Table 1:** Operative mortality with regard to indication and timing*

***Indication***	***n***	***Died***	***Mortality (%)***	***>5 d***
Refractory severe UC	51	8	15	8
Severe LGIH	5	2	40	0
Toxic megacolon	4	2	50	1

* No mortality in perforation and obstruction group

UC: ulcerative colitis; LGIH: Lower gastrointestinal haemorrhage

**Table 2:** Clinical, biochemical and demographic data of the patients who failed intensive medical therapy (*n *= 51) according to the 2 subgroups (Group I: those operated ≤5 days and Group II >5 days after initiation of intensive steroid therapy).

***Variable***	Group I* (n = 17) *Mean (± SEM)*	Group II* (n = 34) *Mean (± SEM)*
Age (years)	33 (3.8)	35 (2)
Disease duration (months)	48 (15.2)	32 (6.7)
Stool frequency/day	10.2 (0.9)	11.9 (0.9)
Pulse rate (/minute)	92 (5.3)	98 (2.5)
Poor nutritional status^#^	9 (56%)	22 (64%)
Hemoglobin (g/dl)	9.9 (0.6)	9.4 (0.4)
ESR (mm)	41 (7.1)	44.8 (4.3)
Urea (mg/dl)	22.2 (1.9)	26.3 (4.8)
Serum albumin (g/dl)	2.8 (0.14)	2.5 (0.13)

* On statistical comparison none of these variables were found to be significantly different

# Number of patients classified as having poor nutritional status

*SEM* - Standard error of means

**Table 3:** The ultimate fate of each patient undergoing emergency sub total colectomy (STC)

***Total patients surviving STC***	**58**
Lost to follow up	3
Refused pouch	2
Pouch not offered	2
	
***Patients eligible for 2^nd ^stage***	**51**
Total IPAA done	46
IRA done	1
TPC	1
Awaiting	3
